# National trends in the use of oral chemotherapy over 13 years

**DOI:** 10.3389/fphar.2022.909948

**Published:** 2022-08-10

**Authors:** A. Moreira, C. Bernardo, C. Ramos, P. Aguiar, F. Alves da Costa

**Affiliations:** ^1^ Medical Oncology Department, Portuguese Oncology Institute of Lisbon Francisco Gentil, Lisbon, Portugal; ^2^ National School of Public Health, Nova University Lisbon, Lisbon, Portugal; ^3^ South-Regional Cancer Registry and Epidemiology Research Unit, Institute of Lisbon Francisco Gentil, Lisbon, Portugal; ^4^ Research Institute for Medicines (iMed.ULisboa), Faculty of Pharmacy, University of Lisbon, Lisbon, Portugal

**Keywords:** oral chemotherapy, consumption data, time trends, hospital oncology, common daily doses, medication adherence

## Abstract

**Background:** Systemic cancer therapy has traditionally been administered using an intravenous (IV) route, implying patients’ frequent visits to hospitals to access to their therapy. If we consider the actual pipeline in oncology, oral chemotherapy will be the main component of cancer treatment in the next few years. This shift in the administration route requires different care models in order to guarantee treatment efficacy and safety.

**Objective:** To analyze time trends in oral chemotherapy consumption in Portugal.

**Method:** Oral chemotherapy consumption over a 13-year period (2008–2020) was analyzed, considering dispensed units by the administration route with respective costs, resorting to the drug regulatory agency (INFARMED I.P.) database. Oral consumption patterns were further explored using common daily doses (CDD) for three conditions, including chronic myeloid leukemia (CML), non-small-cell lung cancer (NSCLC), and breast cancer (BC), to adjust for the effect of varying doses. Data were analyzed descriptively resorting to Microsoft Office Excel 2010.

**Results:** Overall chemotherapy consumption increased +Δ54.7%, with the highest contribution in units observed in oral forms (+Δ58.8%). The total expenditure increased +Δ96.5%, and despite the increase in oral forms (+Δ221.6%), intravenous forms continued to be the major cost driver, with an important contribution from immunotherapy. Much of the increase was led by the approval of 40 new IV and 48 new oral cancer medications with higher costs introduced in the market. Using CDD as an alternative metric to units had varying impacts by indication. The observed increases seemed to focus on specific cancer sites with varying effect; in CML, there was a 2.39-fold increase, compared to 4.41 for NSCLC and 1.86 for BC. However, for BC, two distinct sub-patterns were observed for hormone therapy (increasing 1.83) and for the novel tyrosine kinase inhibitors (increasing 40.8).

**Conclusion:** The growing use of oral chemotherapy is obvious and calls for investments in supporting patients in managing medication adherence and adverse events. The shifts in the healthcare system are complex and need to be prioritized. Our data suggest that priority could be attributed to cancer sites driving innovation, namely, advanced breast cancer.

## Introduction

Systemic cancer treatment has traditionally been administered using an intravenous route, implying that even individuals living independently need frequent visits to hospitals to have access to their treatment. However, in the last decade, the increasing availability of oral anticancer treatments has led patients, families, and clinicians to rethink the existing hospital-centric model of care (Conti et al., 2014; Bosco-Lévy et al., 2017). In fact, the growth of oral anticancer treatments has emerged in the context of increasing survivorship from various tumor types, having in mind the commodity of the individual and avoidance of unnecessary medical care (Jacobs et al., 2017). Contributing to this paradigm shift is the fact that pharmaceutical companies are investing in developing oral counterparts to existing cytotoxic therapies (Azim and Awada, 2012), as well as new oral medications.

The National Comprehensive Cancer Network (NCCN) released a task force report on oral chemotherapy, which found that “the use of oral chemotherapeutic agents profoundly affects all aspects of oncology, including creating significant safety and adherence issues, shifting some traditional roles and responsibilities of oncologists, nurses, and pharmacists to patients and caregivers” (Weingart et al., 2008).

The growing availability of oral anticancer therapies has been explored in different settings, and it has been suggested that it may be particularly relevant in specific groups, namely, in those using targeted therapy (Bosco-Lévy et al., 2017). Previous data on time trends in oncology therapy over the last decade in Portugal showed that the use of oral therapies is increasing over other intravenous therapies. In 2020 in Portugal, according to the National Authority of Medications and Health Products, henceforth referred to as INFARMED I.P., there were 88 different chemotherapy medications marketed in an oral form (Infarmed, 2021).

There are certain cancer types, however, that are particularly appealing to this shift in the administration form, as it is the case of non-small-cell lung cancer (NSCLC) whose treatment is currently targeted at specific mutations, namely, epidermal growth factor receptor (EGFR), anaplastic lymphoma kinase (ALK), ROS proto-oncogene 1, and receptor tyrosine kinase (ROS1) (Geynisman and Wickersham, 2013; Majem and Pallarès, 2013). Chronic myeloid leukemia (CML) constitutes another example where oral treatments have gained a major market share since imatinib’s approval. In breast cancer (BC), the situation is different as hormonal therapy has for long been used in the oral form and even available in ambulatory care, but only since 2018, this form also started to be used in novel cyclin-dependent kinase (CDK) 4/6 inhibitors for advanced breast cancer (ABC), following demonstration of clinical benefit (Finn et al., 2015; Cristofanilli et al., 2016; Rugo et al., 2019). Rising costs of these new therapies are a reason for enhanced attention from payers (Conti et al., 2014), but it is important to consider that consumption increase do not necessarily proportionally translate into costs, above all when focusing on alternative administration forms, which are not equipotent or have equivalent directions of use. Therefore, the use of the traditional consumption metric, the World Health Organization Daily Defined Dose (WHO-DDD) (WHO International Working Group for Drug Statistics Methodology, WHO Collaborating Centre for Drug Statistics Methodology & WHO Collaborating Centre for Drug Utilization Research and Clinical Pharmacological Services, 2003), may not be appropriate to compare different administration routes. DDDs standardize drug use assuming most cases will take the drug for the main indication using the maintenance dose recommended for an adult, which is rarely appropriate in oncology. However, for certain chemotherapy medications, it is possible to estimate that they will be used in one specific cancer type, opening the possibility to explore alternative metrics of consumption.

Following the switch of the preferred administration route and the emergence of new oral medications, this study aims to 1) analyze the time trends in chemotherapy consumption and costs over 13 years in Portugal by the administration route, 2) analyze the effect of using common daily doses (CDDs) or units to measure oral consumption in a specific cancer type (NSCLC), and 3) provide a closer and general evaluation of the drivers of innovation due to the use of CDD in specific cancer types (NSCLC, CML, and BC).

## Materials and methods

A descriptive study was conducted to characterize the trend of chemotherapy medications consumption between 2008 and 2020 (1st January 2008 to 31st December 2020). We resorted to consumption data provided by the INFARMED I.P., reported by publicly administered hospital pharmacies (and there are 45 in the country). INFARMED I.P. is a government agency belonging to the Health Ministry, which is responsible for the evaluation, authorization, regulation, and control of human medicinal products, including medications, medical devices, and cosmetics, with the ultimate goal of protecting public health.

The consumption of chemotherapy medications was estimated in dispensing units (e.g., pills or vials) because it is the metric recommended by the INFARMED I.P. in cases where there is no DDD established.

The analysis was focused on overall time trends, oral route, and intravenous route by units with respective costs per group [tyrosine kinase inhibitors (TKIs), other cytotoxics, hormone therapy, and immunomodulators] and for specific indications. Medications exclusively administered topically and medications without anticancer action were excluded.

As case studies of interest, we have identified substances with a specific indication to further explore trends within those indications considered. For the substances selected as being used for one single indication in ≥ 80% of cases (according to an expert panel of six clinical oncologists), the summary of product characteristics (SmPC) was used to define the most CDD for the specific indication and validated using this same panel. CDD was possible to establish for all oral medications used in NSCLC, CML, and BC (see Table 1).

**TABLE 1:** CDD for medications administered orally for selected indications.

Indication	Medicine	Common daily dose (CDD) for the indication
Non-small-cell lung cancer (NSCL)	Afatinib	40 mg/day
	Alectinib	1,200 mg/day
	Ceritinib	750 mg/day
	Crizotinib	500 mg/day
	Erlotinib	150 mg/day
	Gefitinib	250 mg/day
	Osimertinib	80 mg/day
Chronic myeloid leukemia (CML)	Dasatinib	100 mg/day
	Imatinib	400 mg/day
	Nilotinib	600 mg/day
	Ponatinib	45 mg/day
Breast cancer (BC)	Tamoxifen	20 mg/day
	Letrozole	2.5 mg/day
	Anastrazole	1 mg/day
	Exemestan	25 mg/day
	Palbociclib	125 mg/day
	Ribociclib	600 mg/day
	Abemaciclib	300 mg/day

Subsequently, NSCLC was used as an example, where consumption measured in units and in CDD were compared to evaluate the impact of this alternative metric.

Dispensed units were converted in CDD using the following formula:

$$CDD=\frac{\left(units*w_{dose\;}+units*y_{dose\;}+units*z_{dose\;}+\ldots \right)}{CDD\;defined\;by\;consensus}.$$ (1)

Caption: w_dose_, y_dose_, and z_dose_ correspond to the diverse possible available doses of the medicine.

Variation (%) was also calculated using the formula: $\frac{x_{final\;x100\%}}{x_{initial}}.$

The difference in the amplitude of effect was presented as a percent value, and the relative contribution of each drug in each time point was also observed to verify if a linear trend could be observed.

A similar exercise was replicated for the other two indications, BC and CML, and the consumption measured in CDD during the study period was estimated for the three indications. Data were analyzed using Microsoft Excel 2010.

This study was exempted from ethics approval because public data were made available by INFARMED I.P. at an aggregate population level, not enabling the identification of individual consumption.

## Results

A total of 194 medications were analyzed, of which 119 were available for administration intravenously and 88 available for administration orally (13 medications were counted twice as they were available for administration in both routes). Moreover, during this period, a total of 88 new medications were approved in the market, 48 in the oral form and 40 in the intravenous form. There were 13 medications available for both routes of administration. Most of the analyzed medications belong to the pharmacotherapeutic group *other cytotoxics* (*n* = 86), followed by *immunomodulators* (*n* = 50).

### Anticancer consumption trend per administration route (oral and intravenous routes)

**FIGURE 1:**
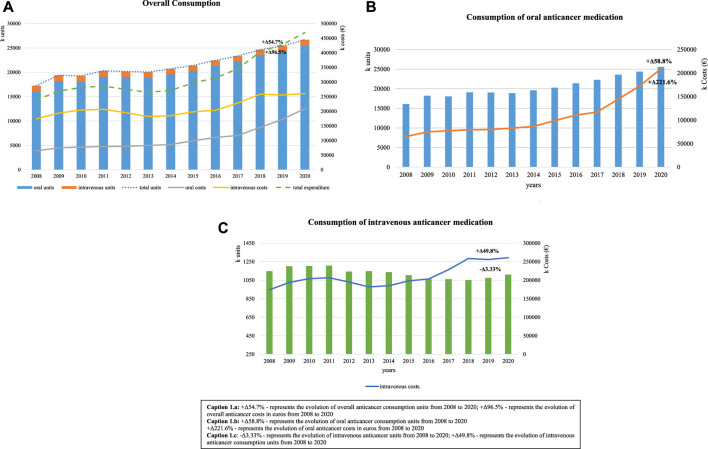
Consumption trends of anticancer medication over a 13-year period, measured in units with respective costs. **(A)** +Δ54.7% represents the evolution of overall anticancer consumption units from 2008 to 2020 and +Δ96.5% represents the evolution of overall anticancer costs in euros from 2008 to 2020. **(B)** +Δ58.8% represents the evolution of oral anticancer consumption units from 2008 to 2020 and +Δ221.6% represents the evolution of oral anticancer costs in euros from 2008 to 2020. **(C)** −Δ3.33% represents the evolution of intravenous anticancer units from 2008 to 2020 and +Δ49.8% represents the evolution of intravenous anticancer consumption units from 2008 to 2020.

During this 13-year study period, an increase was observed in overall consumption of anticancer medications, from 17,250,308 units in 2008 to 26,687,335 units in 2020 (Δ +54.7%) (Figure 1A). Oral anticancer registered an increase of Δ +58.8% (16,102,410 units to 25,577,613 units). Expenditure in oral anticancer registered in 2008 was 64,929,848€, and it increased constantly until 2020, when 208,808,013 € were recorded (Figure 1B). Intravenous forms recorded a decrease in consumption (Δ −3.33%; from 1,147,899 units to 1,109,722 units), albeit the costs continuing to increase, corresponding to an additional 260,643,443€ spent in 2020, 49.8% more than that in 2008 (Figure 1C). However, the cost share of intravenous medication decreased throughout the period, from 72.8% in 2008 to 55.5% in 2020, still representing most of the expenditure of anticancer medications.

### Oral consumption trend per pharmacotherapeutic group with respective costs

**FIGURE 2:**
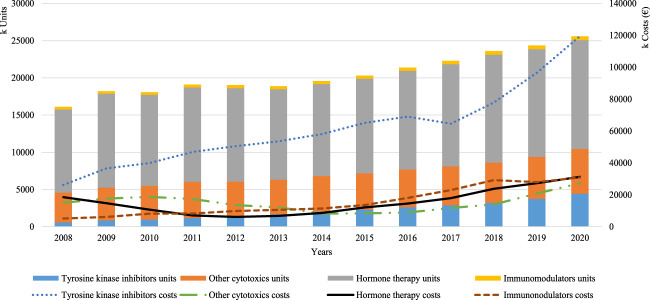
Overall consumption per pharmacotherapeutic group.

Observing closely the pharmacotherapeutic groups that mostly drove this increase in the oral forms, we can see that hormone therapy was the subgroup representing a larger proportion of consumption throughout the study period, with a total of 11,174,108 units consumed in 2008 and 14,629,223 units in 2020 (Figure 2). However, the pharmacotherapeutic group representing the highest increase in consumption and also the higher costs over the period of analysis were the TKIs, with a recorded consumption increase of 676.4%, with varying corresponding costs between 40.3% (in 2008) and 67.2% (in 2014) as share of the total oral anticancer expenditure.

### A snapshot of oral medications used in non-small-cell lung cancer using two alternative metrics of consumption

**FIGURE 3:**
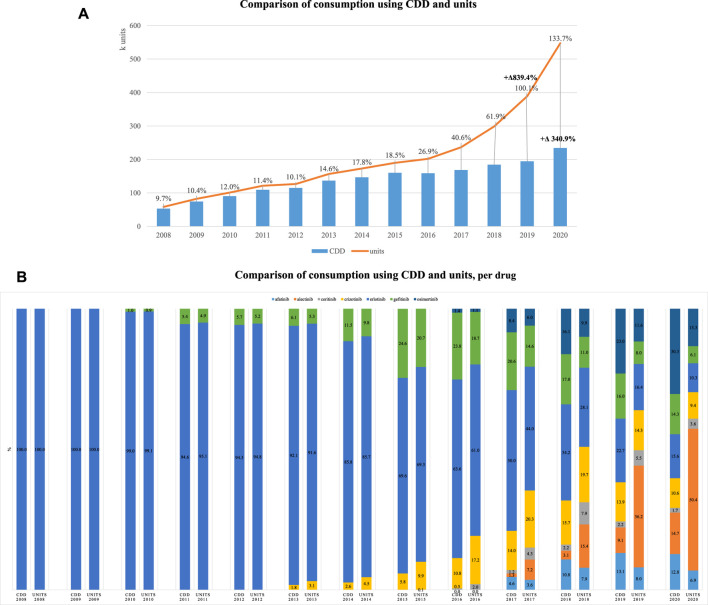
Consumption of oral medications used for NSCLC. **(A)** Comparison of consumption using CDD and units. **(B)** Comparison of consumption using CDD and units per drug.

Using NSCLC as a case study of interest, oral consumption through the study period increased 839.5% measured in units and only 340.9% measured in CDD; the latter suggesting being a more accurate measure to capture changes in consumption patterns that also consider dose adaptations (Figure 3A). Focusing on the different medications used for this indication, we could observe that in the first 2 years, measurement in units and in CDD was equivalent as only erlotinib was available, but as more therapeutic options became available in oral forms, the use of different metrics had varying impacts on estimates of within year proportion. For example, in 2020, alectinib recorded a consumption corresponding to 14.7% and 50.4%, respectively, for CDD and units. Conversely, afatinib in 2017 had very similar consumptions, regardless of metric, 4.6% and 3.6% (Figure 3B).

This same exercise was replicated for BC, but no visible effects were observed, with consumption increase of 89.8% and 88.2%, respectively, for units and CDD. However, restricting the analysis to CDK 4/6 inhibitors, the disproportionate effect of the metric also became visible, increasing 6414.1% and 3981.1%, respectively, for units and CDD, between 2017 and 2020.

### Analyzing consumption (in common daily doses) of oral medications for specific cancer sites: breast cancer, chronic myeloid leukemia, and non-small-cell lung cancer

**FIGURE 4:**
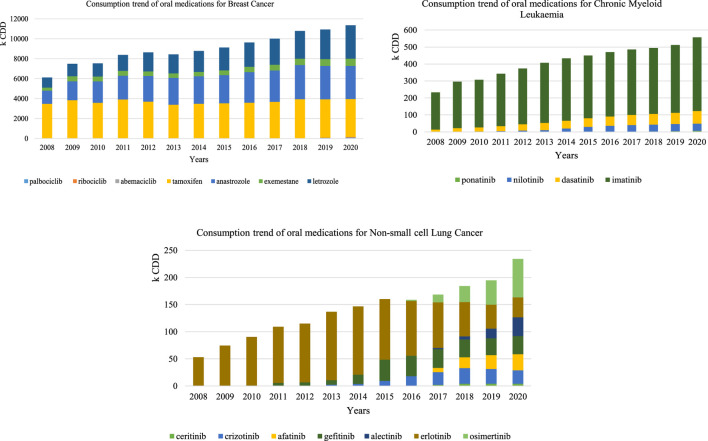
Consumption trend using the CDD of oral anticancer for breast cancer, chronic myeloid leukemia, and non-small-cell lung cancer

Based on previous findings, CDD were therefore considered the best metric to observe time trends for specific indications, particularly when adjustment is needed to take into account the fact that the standard dose results from combining various marketed units, and therefore was used for the three case studies of interest (Figure 4).

Oral medications with a CML indication had a 2.39-fold increase in consumption (232,662 CDD in 2008 and 556,966 CDD in 2020). Imatinib was the most consumed medicine over the study period, registering 218,818 CDD in 2008 and 434,263 CDD in 2020, followed by dasatinib that constantly increased (12,863 CDD in 2008 and 73,801 CDD in 2020).

Oral consumption of medications with NSLC indication registered a 4.41-fold increase (from 53,132 CDD in 2008 to 234,250 CDD in 2020). Erlotinib represented the total consumption between 2008 and 2009, as it was the only available oral form then. From 2010 onwards, new oral forms were introduced and osimertinib consumption progressively gained more preponderance, being the most consumed medicine for this indication in 2020.

Oral medications with a BC indication had a 1.86-fold increase overall from 2008 to 2020. However, broken down by the pharmacotherapeutic group, the increase in hormone and adjuvant therapy exclusively corresponded to a 1.83-fold increase, whereas the increase recorded for CDK 4/6 inhibitors between 2017 and 2020 was 40.8 fold.

Our data showed an increase in overall anticancer consumption of +∆54.7% between 2008 and 2020. The net relative increase observed for oral forms was 7.5% higher than the overall increase (58.8%/54.7%). Overall costs of therapies with anticancer medications increased from 2008 to 2020 by +∆96.5%. The major change was observed in costs of oral forms, which have increased +∆221.6%, apparently driven by the introduction of new and more costly therapies. The overall cost increase does not seem to result from the shift in the administration form, since intravenous route continues to represent the main proportion of the expenditure in anticancer consumption, representing 55.5% up to 72.8% of the overall consumption. The hormone therapy pharmacotherapeutic group registered the major proportion of oral consumption over this period, which is not surprising considering most of their use is for BC, the most incident cancers among women (International Agency for Research on Cancer G, 2020). Notwithstanding, TKIs recorded the sharpest increase and represented most of the expenditure, which may be explained by the introduction of new oral forms, +∆64.5% of which are TKIs (*n* = 31). This may be explained by several new targets that this class has action on, thus allowing medicines optimization.

The proposed alternative consumption measure aims to overcome some of the limitations of assessing consumption using dispensed units, mostly when ascertaining different routes as comparing a vial with a pill may be misleading. Most medicines administered in oncology do not have an attributed DDD given their different indications. The observed switch in the administration route justifies the need for a new metric of drug consumption which adjusts for dosage and indication. The CDD proposed in this study are restricted to specific indications and oral forms. Nonetheless, there are studies proposing other metrics of drug consumption (WHO International Working Group for Drug Statistics Methodology, WHO Collaborating Centre for Drug Statistics Methodology & WHO Collaborating Centre for Drug Utilization Research and Clinical Pharmacological Services, 2003).

For the metric to be operationalized, we need to consider that the frequency of administration of oral and intravenous medications (e.g., most oral forms require taking daily units whereas intravenous administration may be weekly or monthly), as well as their potency, are different and influence consumption data. Moreover, the choice of therapy in oncology requires multidisciplinary practices with frequent adaptation of doses during the course of therapy (Shulman, 2015) and in some particular pharmacotherapeutic groups requiring the use of multiple units, therefore requiring adjustments. As data suggested that oral forms are driving innovation, it seemed reasonable to focus our attention on oral forms. Our results of afatinib and alectinib illustrate this idea very well, as the first is available on the market with a dose corresponding to the daily recommended dose in NSCLC, that is, one pill per day, whereas alectinib is available as 150 mg and the recommended dose for this indication is 1,200 mg, implying eight pills (units) need to be taken daily. Therefore, comparing consumption in CDD and in units, using NSCLC as a case study of interest, suggested that CDD could be a better metric to adjust for the effect of varying doses used by indication.

Analyzing consumption for the three specific indications, a clear increase in the use of oral forms was observed over the study period. TKIs were a possible treatment for all selected indications as treatment, with a constant increase aside the introduction of new medications. However, the increase was not proportional across the three indications, with the lowest observed in BC (1.86-fold increase), followed by CML (2.39-fold increase), and finally NSCLC (4.41-fold increase), suggesting lung cancer is attracting much of the innovation. Imatinib’s constant increase in consumption observed is likely to result from being the first line treatment for CML, justified by demonstrated gains in 10-year overall survival (Hochhaus et al., 2017). The 4-fold increase in NSCLC medications seems to be driven by the introduction of new TKIs to overcome erlotinib’s acquired resistance mechanisms, including gefinitib, osimertinib, afatinib, crizotinib, alectinib, and ceritinib (Zhong et al., 2017). However, taking a closer look at BC, we could identify two distinct patterns, where a modest increase corresponded to hormone therapy and a very sharp increase caused by TKIs more recently introduced for ABC. These were not so visible initially as hormone therapy represent an enormous proportion of consumption, justified by previous literature defending hormone therapy to be more cost-effective than classical anticancer in BC patients (Diaby et al., 2015). CDK 4/6 inhibitors were introduced in 2017 for ABC; first represented by palbociclib and soon followed by ribociclib in 2018 and abemaciclib in 2020. Considering only this subgroup, the increase was 40.8 fold, supported by evidence from clinical trials and more recent real-word studies (Cardoso Borges et al., 2022).

Ours results give clear information about consumption of several drugs in specific types of cancer and allow clinicians to understand which are the current medicines that have been used to treat these conditions. In our opinion, our study provides valuable information for clinical practice as the use of precise data consumption in this area may help, for example, researchers to implement pharmacovigilance or effectiveness studies. The traditional approach to cancer care focuses very much on the use of the intravenous route, with the patient needing to rely on a healthcare professional to administer it. This current shift in the administration form calls for a reorientation of healthcare organization and even financing, so that patients may become accountable for medication adherence and are supported by healthcare professionals in managing the adverse effects of their medication. This analysis is mainly descriptive but raises issues around health policy and calls for investments being made, particularly in areas driving costs, such as ABC, where surely women need to be supported in their choices during their survivorship. Also, financing mechanisms need to be revisited taking this new paradigm into account as currently the financing model of hospitals in many European countries only considers costs of procedures and therefore “administration of oral forms” does not get imputed. Other aspects worth emphasizing are equity and commodity, which should favor patients’ autonomy to obtain medication in ambulatory care, through community pharmacies or primary care clinics. However, this assumes that the same medication cannot be also available at hospital and with a different reimbursement policy, as this possibility will have perverse results that potentially impact even on medication adherence.

In our view, this study has the main strength of overcoming limitations in drug utilization studies by considering a new metric. However, there are some limitations worth acknowledging, namely the fact that data used represents exclusively consumptions from the public sector. In Portugal, there are currently 225 hospitals, among which 118 are private^20^. However, we considered this aspect of minimal importance since only a minority of private structures includes oncologic therapy.

## Conclusion

A clear increase in oral anticancer medications was visible in the study period, aligned with increased costs for healthcare systems. The implications for practice reorientation may be that greater commodity and independence for oncology patients creates a demand for remote assistance and restructuring of healthcare services, to better assist people in managing adverse events and medication adherence.

## Data Availability

The raw data supporting the conclusion of this article will be made available by the authors, without undue reservation.
